# Lingguizhugan Decoction in the Treatment of Non-Alcoholic Fatty Liver Disease: A Systematic Review and Meta-Analysis

**DOI:** 10.2174/0118715303323071241022053842

**Published:** 2025-01-09

**Authors:** Yifan Lu, Lijuan Nie, Xinyi Yang, Ziming Zhao, Yuxiao Wang, Qibiao Wu, Xiqiao Zhou

**Affiliations:** 1 State Key Laboratory of Quality Research in Chinese Medicines, Faculty of Chinese Medicine, Macau University of Science and Technology, Macau, P.R. China;; 2 The First Clinical Medical College of Nanjing University of Chinese Medicine, Nanjing City, Jiangsu Province, 210000, China;; 3 Department of Endocrinology, Jiangsu Province Hospital of Chinese Medicine, Affiliated Hospital of Nanjing University of Chinese Medicine, Nanjing City, Jiangsu Province, 210000, China

**Keywords:** Ling Gui Zhu Gan Decoction, non-alcoholic fatty liver disease, systematic review, meta-analysis, traditional Chinese medicine, controlled trials

## Abstract

**Objective:**

This study systematically evaluated the efficacy and safety of Ling Gui Zhu Gan Decoction for treating non-alcoholic fatty liver disease.

**Methods:**

Registered under CRD42024501460 on the PROSPERO platform, we searched eight major databases, including Web of Science, PubMed, Cochrane, Embase, China National Knowledge Infrastructure, Wanfang database, Chinese Scientific Journals Database, and Chinese Biomedicine Database, from inception to December 2023 for randomized controlled trials on Ling Gui Zhu Gan Decoction in non-alcoholic fatty liver disease treatment. We extracted data on total efficiency, TC, TG, ALT, AST, GGT, and HOMA-IR, analyzing results with RevMan 5.4 software.

**Results:**

Twelve studies met the inclusion criteria, encompassing 970 cases. Ling Gui Zhu Gan Decoction, alone or combined with standard therapy, significantly improved non-alcoholic fatty liver disease outcomes, regardless of treatment duration. Only one study reported adverse events, including bloating, diarrhea, nausea, vomiting, and rash.

**Conclusion:**

Ling Gui Zhu Gan Decoction appears to be an effective and safe option for non-alcoholic fatty liver disease treatment. However, due to limited studies and methodological weakness, further rigorous randomized controlled trials are necessary for conclusive results.

## 1 INTRODUCTION

Non-alcoholic Fatty Liver Disease (NAFLD) is characterized by excessive fat accumulation in the liver without significant alcohol use. It ranges from simple fatty liver (steatosis) to non-alcoholic steatohepatitis (NASH), which involves liver inflammation and damage [1]. NAFLD is the most common chronic liver disease globally, affecting approximately 25% of the population, and is increasingly prevalent due to obesity, type 2 diabetes, and metabolic syndrome [2]. Projections indicate that NAFLD may soon surpass hepatitis C as the leading cause of liver transplantation [3]. Progressive fibrosis, cirrhosis, and liver failure are serious complications of NAFLD, with NASH being more severe due to its inflammatory nature [4, 5]. The presence of fibrosis is a key predictor of poor outcomes in NAFLD. Cardiovascular disease is a leading cause of death among NAFLD patients, and the disease itself heightens heart disease risk [3].

The pathogenesis of NAFLD is often explained by the “double whammy” hypothesis, which posits a multifactorial interplay of factors, including lipotoxicity, activation of the innate immune system, genetic predispositions, and microbiome influences along with environmental factors. This hypothesis suggests that the progression from simple steatosis to NASH requires an additional contributing factor, such as oxidative stress [6]. Despite advances in understanding the mechanisms underlying hepatic steatosis, the complete pathogenesis of NASH remains incompletely understood. Numerous molecular pathways are involved in the progression of NASH, and the causative drivers of the disease vary among patients, leading to significant heterogeneity in the mechanisms and clinical manifestations [7]. Key factors influencing NASH include lipotoxicity, endoplasmic reticulum (ER) stress, mitochondrial dysfunction, oxidative stress, intestinal endotoxins, and microbiota [8-12]. Lipotoxicity arises when fatty acid supply exceeds processing capacity, potentially triggering ER stress, hepatocyte injury, inflammation, and fibrosis [13].

Diagnosing NAFLD involves identifying the presence of steatosis while excluding other secondary factors, such as alcohol consumption or drug use, and conducting risk stratification for NASH and fibrosis [3]. Liver biopsy is a valuable diagnostic tool for assessing NASH and fibrosis in patients deemed at significant risk based on non-invasive markers that help differentiate between simple fatty liver and NASH.

Management of cardiovascular risk factors, such as blood pressure, lipid levels, body weight, smoking cessation, and assessment and management of diabetes mellitus, is emphasized as a preventive strategy and primary care approach for NAFLD [1]. Evidence indicates that weight loss from diet and exercise reduces liver fat content. It also enhances insulin resistance and homeostasis in human adipose tissue [14]. However, sustained lifestyle changes are challenging for many patients. As a result, some may seek pharmacological interventions. The field of pharmacological treatment for NASH is advancing rapidly. As of March this year, no drugs have been approved for treating NAFLD. Promising options include vitamin E, which combats oxidative stress and maintains the intracellular redox state [15]. Farnesoid X-receptor agonists, such obeticholic acid, improve insulin resistance and have direct anti-inflammatory and anti-fibrotic effects. Peroxisome proliferator-activated receptor-α/δ agonists (*e.g.,* pioglitazone) can ameliorate steatosis, inflammation, and hepatic fibrosis. They also improve insulin resistance and hepatic enzyme levels [16, 17]. Inhibitors of C-C chemokine receptors 2 and 5 enhance the innate immune response of the liver and reduce short-term fibrosis progression [18]. Other notable agents include THRβ agonists, CR2/5 antagonists (*e.g.,* 27-cenicriviroc), and ASK1 inhibitors (*e.g.,* selonsertib) [1]. Glucagon-like peptide-1 receptor agonists (GLP-1RA), such as liraglutide, are approved for diabetes and have shown initial effectiveness in NASH. Selective sodium-dependent glucose co-transporter 2 (SGLT2) inhibitors also demonstrate potential cardiovascular and renal benefits [19]. Existing drug treatments for NAFLD target several pathogenic mechanisms. This excess lipid accumulation, disturbed redox homeostasis, compromised mitochondrial function, and liver architectural remodeling [15]. However, current medical approaches, such as medications for diabetes, dyslipidemia, and bile acid regulation, have shown limited effectiveness in halting disease progression [20, 21]. Most therapeutic agents have produced unsatisfactory results in clinical trials, with liver histological endpoints not yet achieved [22]. The treatment of NAFLD and its progressive form, NASH, remains challenging. NASH and fibrosis can lead to cirrhosis and further clinical complications. Current treatments either aim to attenuate metabolic dysregulation and cellular damage or directly target inflammation and fibrosis [23].

Before March of this year, there was no FDA-approved potent drug for the treatment of NASH. However, in March, the U.S. Food and Drug Administration (FDA) announced the approval of Rezdiffra, an oral small molecule drug with the active ingredient resmetirom. This drug is specifically for metabolic dysfunction-associated steatohepatitis in patients with intermediate to advanced hepatic scarring (fibrosis). Resmetirom is a selective agonist for the thyroid hormone receptor-β. Activating this receptor in the liver helps reduce hepatic fat accumulation. An article published in February reported on phase 3 clinical trial assessing the efficacy of resmetirom in adults with NASH and various stages of fibrosis. The results indicated that patients treated with 80mg and 100mg of resmetirom showed greater regression of NASH and improvement in at least one stage of hepatic fibrosis compared to placebo [24]. Additionally, a study in the journal *Nature* highlighted the positive efficacy of it in treating NASH with liver fibrosis [25].

Chinese medicine compounds have a pharmacological effect through multi-component, multi-pathway, and multi-target integrated interventions, which can help alleviate NAFLD and its related complications [26]. Advances in network pharmacology identified 10 core candidate targets of Ling Gui Zhu Gan Decoction (LGZG): ALB, TNF, IL6, AKT1, PPARG, VEGFA, EGFR, ESR1, SIRT1, and STAT3. These targets are associated with toxic metabolite accumulation, antioxidant activity, inflammation, glucose-lipid metabolism disorders, hepatocellular carcinoma, lipid metabolism, hepatic injury, and mitochondrial energy metabolism. All 10 targets show good binding to the top 20 active ingredients of LGZG and exhibit potential pharmacological functions through 10 major pathways related to cancer, lipid and atherosclerosis, diabetic cardiomyopathy, and insulin resistance [27]. LGZG consists of four Chinese herbs and originated from *Shanghan Zabing Lun* (Table **1**). It is a well-known traditional Chinese medicine (TCM) formula that has been clinically used to treat NAFLD. Studies indicate that LGZG can be combined with other drugs or dietary interventions for treating NAFLD. Experimental research suggests that LGZG may influence mitochondrial energy metabolism, regulate lipid metabolism disorders, and reduce inflammatory injury in hepatocytes. While the efficacy of LGZG has been confirmed by various clinical and experimental studies, a systematic evaluation of this combination has yet to be conducted. Therefore, considering LGZG as a promising alternative therapy, we conducted this meta-analysis to provide systematic evidence supporting its use in treating NAFLD.

## 2 METHODS

This systematic review and meta-analyses was conducted following the PRISMA (Preferred Reporting Items for Systematic Reviews and Meta-Analyses) guidelines.

### 2.1 Search Strategy

A comprehensive literature search was performed across multiple databases from inception through December 2023, including Web of Science, PubMed, Cochrane Central Register of Clinical Trials, Embase, China National Knowledge Infrastructure (CNKI), Wanfang Database, Chinese Scientific Journals Database (VIP), and Chinese Biomedicine Database (CBM). The search strategy is summarized in Table **2**, using Web of Science as an example.

### 2.2 Inclusion Criteria

Trials eligible for inclusion were:

1. Parallel-group randomized controlled trials (RCTs) enrolling patients diagnosed with NAFLD according to applicable clinical practice guidelines [28].
2. Studies evaluating LGZG alone or in combination with standard therapy compared to standard therapy alone. Standard therapies included pharmaceutical, lifestyle, or dietary interventions [29].
3. Trials reporting at least one outcome measure, including total effective rate (TER), total cholesterol (TC), triglycerides (TG), alanine aminotransferase (ALT), aspartate aminotransferase (AST), glutamyl transpeptidase (GGT) [30], or homeostatic model assessment of insulin resistance (HOMA-IR).

### 2.3 Exclusion Criteria

Studies were excluded if they:

1. Deviated from the central research focus.
2. Were not published in English or Chinese.
3. Were reviews, editorials, case reports, and commentaries [29].

### 2.4 Data Extraction and Risk of Bias Assessment

Two authors independently extracted relevant information into a table, including the number of cases, age, sex, disease duration, interventions, treatment duration, follow-up, and outcome indicators. The methodological quality of the literature was assessed using the Cochrane Risk of Bias Assessment Tool across seven areas: random sequence generation, allocation concealment, blinding of participants and researchers, blinding of outcome data, outcome data completeness, selective reporting, and other biases. A study was rated as having a low risk of bias if all aspects were assessed as low, high risk if any aspects was high, and uncertain risk if no aspects were assessed as high, but some were uncertain. Disagreements were resolved through discussion with a third author.

### 2.5 Statistical Analysis

Data were analyzed using RevMan 5.4 software. We calculated the WMD for dichotomous and continuous variables with 95% CIs. Based on I^2^ values and *P* values, we selected appropriate effect models: a fixed-effect model for I^2^<50% and a random-effect model for I^2^>50%. Meta-analysis was conducted without heterogeneity, and forest plots were generated for observed indicators. Funnel plots assessed potential publication bias, and subgroup analyses were performed for a comprehensive evaluation of treatment duration.

### 2.6 Ethics

As all the studies included in the analysis were already published, no further ethical approval was required.

## 3 RESULTS

### 3.1 Characteristics of Included Trials

**Fig. (1):**
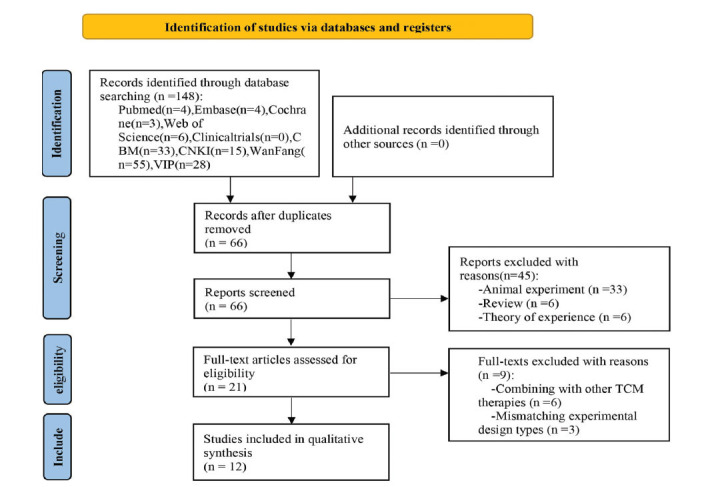
Flow diagram of literature screening.

A total of 148 relevant articles were retrieved. After removing duplicates based on title and publication date, 66 articles remained. Upon reviewing the abstracts, we exclude literature focused on mechanism studies, empirical theories, and reviews, leaving 21 randomized controlled trials (RCTs). Following a thorough evaluation of the abstracts and full texts and applying inclusion and exclusion criteria, 12 articles [30-41] were ultimately included for data analysis (Fig. **1**).

In total, 970 individuals participated across the 12 articles, with the experimental group consisting of 489 participants and the control group comprising 481 participants. All articles were published in Chinese. Ten studies compared LGZG combined with basic treatment to basic treatment alone, while the remaining studies compared LGZG with basic treatment. Basic treatment included Western medicine, physical exercise, or a healthy diet. Treatment durations ranged from 20 days to 3 months, with disease duration between 2.5 and 8.47 years. Three studies reported a follow-up period of 4 to 24 weeks. One study involving polyene phosphatidylcholine capsules noted adverse events, including bloating, diarrhea, nausea, vomiting, and rash (Table **3**).

One study used ultrasonography to assess hepatic steatosis as the criterion for efficacy. Six studies relied on Chinese medicine evidence scores before and after treatment for efficacy assessment, while four studies used symptoms, signs, ALT, AST, TG, and ultrasound as criteria.

### 3.2 Methodological Quality of the Included Trials

**Fig. (2):**
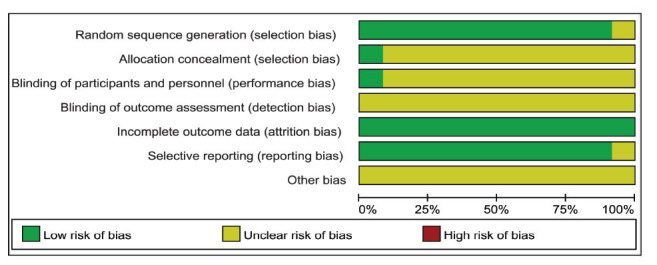
Risk of bias graph.

**Fig. (3):**
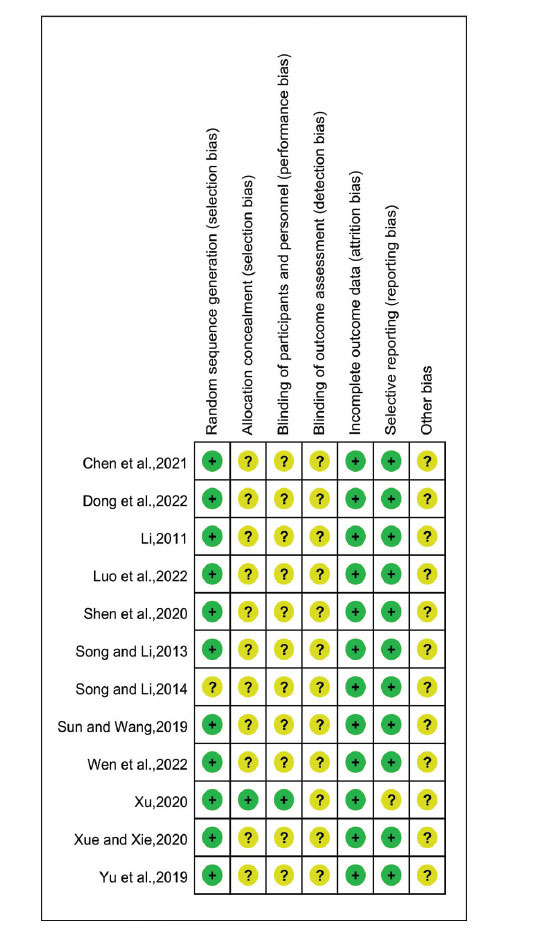
Risk of bias summary. Study 1-12 [30-41].

Random assignment was reported in all studies included in the analysis, except for one study [39] that did not specify a randomization method, such as a random number table. Only one study [36] indicated the use of allocation concealment and blinding for both subjects and researchers. Additionally, one study [36] had incomplete data due to a participant withdrawing midway. Due to insufficient information on the risk of blinding, we categorized the risk in these studies as uncertain. Overall, all 12 studies were rated as having unclear risk (Figs. **2** and **3**) [29].

### 3.3 Outcome Measures

#### 3.3.1 Total Effective Rate of LGZG

**Fig. (4):**
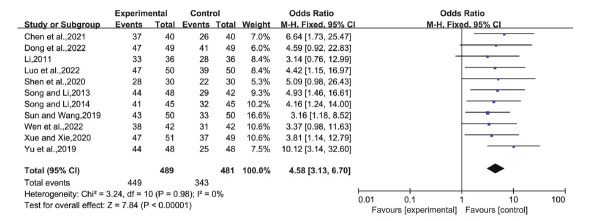
Total effective rate of LGZG with an essential treatment *versus* an essential treatment. **Abbreviation:** LGZG= Lingguizhugan Decoction.

Eleven studies compared LGZG with or without basic treatment to basic treatment alone. The analysis showed no significant heterogeneity (I^2^=0%, P=0.98), so we adopted a fixed-effects model. The odds ratio (OR) was 4.58 (95% CI: 3.13, 6.70), *P* < 0.00001, indicating a significant difference between the trial and control groups (Fig. **4**). This suggests that the overall efficacy of LGZG in treating NAFLD is superior to that of basic therapy alone.

#### 3.3.2 Serum Biochemical Indices of NAFLD

**Fig. (5):**
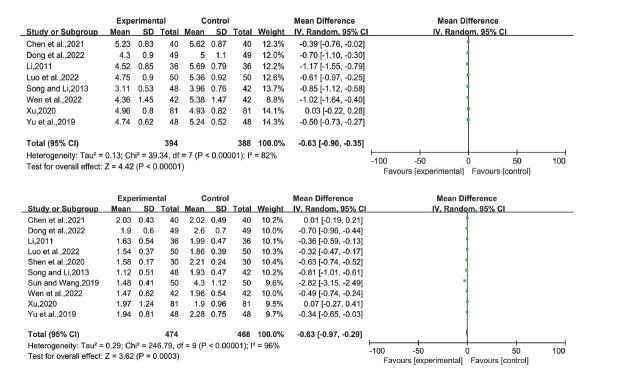
TC and TG of LGZG *versus* an essential treatment. **Abbreviations:** LGZG= Lingguizhugan Decoction, TC= Total cholesterol, TG= Triglyceride.

**Fig. (6):**
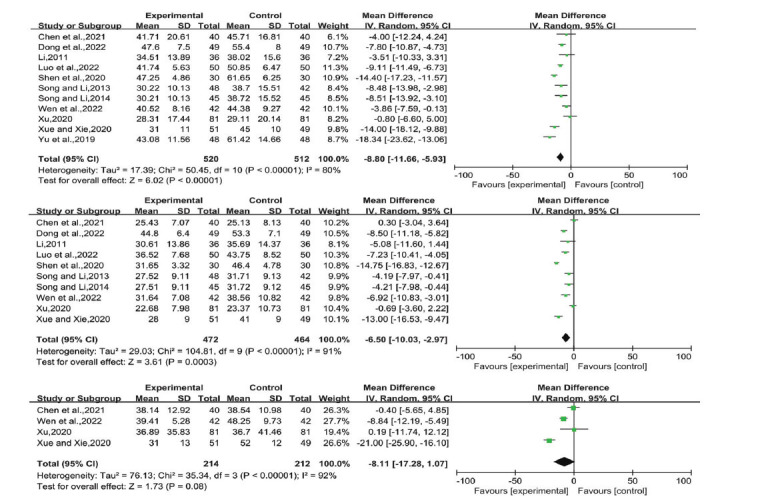
ALT, AST, GGT of LGZG *versus* an essential treatment. **Abbreviations:** LGZG= Lingguizhugan Decoction, ALT= Alanine aminotransferase, AST= Aspartate aminotransferase, GGT= Glutamyl transpeptidase.

**Fig. (7):**
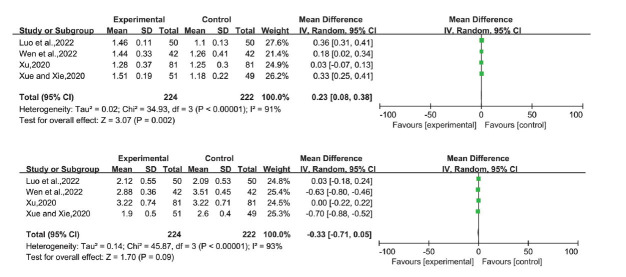
HDL-C, LDL-C of LGZG *versus* an essential treatment. **Abbreviations:** LGZG= Lingguizhugan Decoction, HDL-C= High-density lipoprotein cholesterol, LDL-C= Low-density lipoprotein cholesterol.

**Fig. (8):**
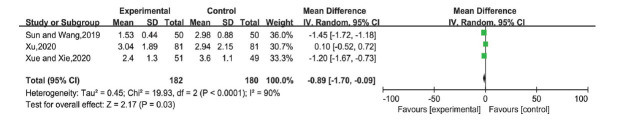
HOMA-IR of LGZG *versus* an essential treatment. **Abbreviations:** LGZG= Lingguizhugan Decoction, HOMA-IR= Homeostasis model assessment.

Eight studies reported TC levels, ten reported TG levels, eleven reported ALT levels, ten reported AST levels, four reported HDL-C, LDL-C, and GGT levels, and three reported HOMA-IR levels. Based on the *P*-value and I^2^ values, we used a random-effects model. The mean differences (MD) were as follows (Figs. **5**-**8**):

TC: -0.63 (-0.90, -0.35), *P* < 0.00001

TG: -0.63 (-0.97, -0.29), *P* = 0.0003

ALT: -8.80 (-11.66, -5.93), *P* < 0.00001

AST: -6.50 (-10.03, -2.97), *P* = 0.0003

GGT: -8.11 (-17.28,1.07), *P* = 0.08

HDL-C: 0.23 (0.08,0.38), *P* = 0.002

LDL-C: -0.33 (-0.71,0.05), P=0.09

HOMA-IR: -0.89 (-1.70,-0.09), *P* = 0.03

Results for all indicators except LDL-L and GGT suggest a notable distinction between the experimental and control groups, indicating that LGZG is superior to basic therapy in regulating lipids and protecting liver function.

#### 3.3.3 Total Effective Rate Based on Disease Duration

**Fig. (9):**
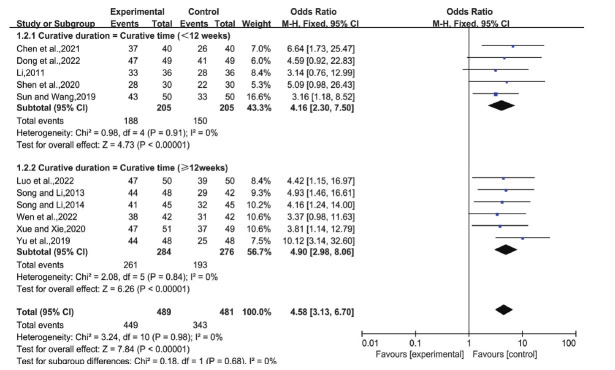
Total effective rate for different curative duration of NAFLD. **Abbreviations:** NAFLD= Non-alcoholic fatty liver disease.

Subgroup analyses were conducted based on treatment duration: ≥12 weeks (longer) and <12 weeks (shorter). Six studies fell into the longer duration category, while five were in the shorter category. The analysis showed no significant heterogeneity (I^2^=0%, *P*=0.91 for longer; I^2^=0%, *P*=0.84 for shorter), prompting the use of a fixed-effects model. The ORs for longer and shorter durations were 4.16 (95% CI: 2.30,7.50), *P*<0.00001, and 4.90 (95% CI: 2.98,8.06), *P*<0.00001, respectively, signifying a significant difference between groups (Fig. **9**). This suggests that LGZG is effective in treating NAFLD across different durations.

### 3.4 Adverse Events

One study [35], which combined LGZG with polyene phosphatidylcholine capsules, reported 13 adverse events see (Table **3**). In the treatment group, there was one case of bloating, one case of diarrhea, two cases of nausea, one case of vomiting, and one case of rash. In the control group, there were two cases of bloating, one case of diarrhea, two cases of nausea, one case of vomiting, and one case of rash. These events may be associated with the polyene phosphatidylcholine capsules, requiring further investigation for validation. Overall, documentation of adverse events was insufficient, highlighting the need for more research on the safety of LGZG for NAFLD.

### 3.5 Sensitivity Analysis

The sensitivity of this meta-analysis was assessed by manually excluding studies one at a time and observing changes in risk ratios (RR) or weighted mean difference (WMD). Results indicated that excluding the study by Xu Jingjuan [36] from the analyses of HDL-C and HOMA-IR showed mild effects on heterogeneity (I^2^=57%, *P*=0.10 for HDL-C; I^2^=0%, *P*=0.37 for HOMA-IR), which remains within acceptable limits. The WMD for the remaining indicators showed little variation, suggesting that the results are credible.

### 3.6 Publication Bias

**Fig. (10):**
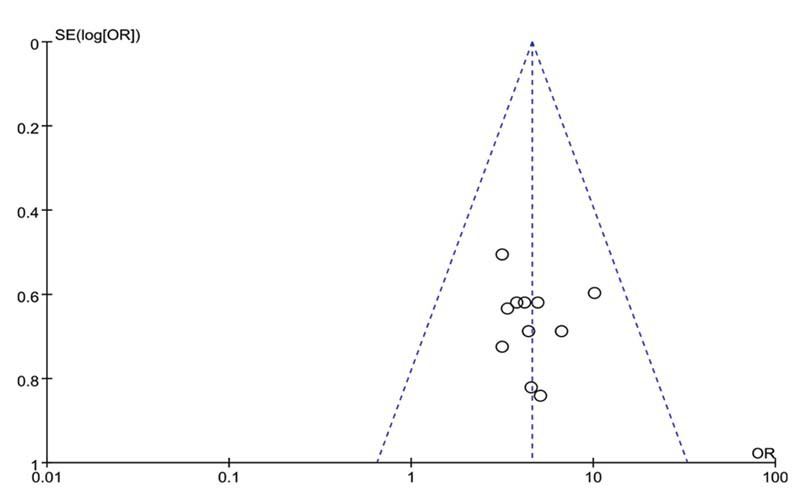
Funnel plot of total effective rate of LGZG for NAFLD. **Abbreviations:** LGZG= Lingguizhugan Decoction, NAFLD= Non-alcoholic fatty liver disease.

**Fig. (11):**
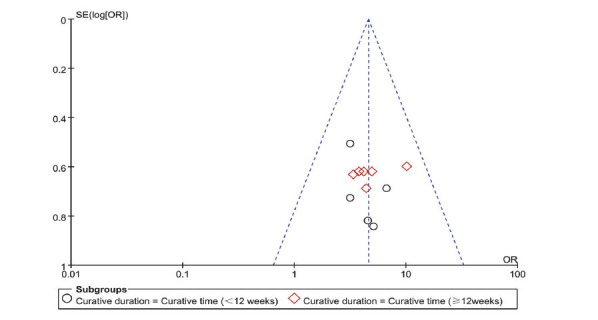
Funnel plot of total effective rate for different curative duration of LGZG for NAFLD. **Abbreviation:** LGZG= Lingguizhugan Decoction, NAFLD= Non-alcoholic fatty liver disease.

Funnel plots were used to analyze the total effective rate and the total effective rate for different treatment durations. The symmetry of the plot suggests a lower likelihood of publication bias (Figs. **10** and **11**).

## 4 DISCUSSION

In traditional Chinese medicine (TCM), NAFLD lacks a specific designation. However, it is often classified based on its etiology and symptoms as “Gan Pi”, “Xie Tong”, “Tan Zheng”, or “Tan Zhuo”. The prevailing pathogenesis involves liver depression and spleen deficiency, alongside internal phlegm, dampness, and stasis accumulation. LGZG, containing Poria, Cassia Twig, Atractylodes Macrocephalae, and Liquorice Root, is a key formula for NAFLD treatment. Modern medicine defines NAFLD as diffuse hepatocellular steatosis, with causes linked to insulin resistance, lipid peroxidation, intestinal dysbiosis, endoplasmic reticulum stress, adipose tissue dysfunction, mitochondrial issues, autophagy, and various genetic and environmental factors. Pharmacological studies demonstrate that Poria cocos improves insulin resistance, reduces oxidative damage, and regulates lipid metabolism [42, 43]. LGZG has shown efficacy in decreasing hepatic lipid deposition, improving liver histopathology, regulating oxidative stress [35], managing hepatic glycogen metabolism [44], and alleviating insulin resistance and inflammation [45, 46].

This meta-analysis reveals that LGZG significantly improves ALT, AST, TC, TG, HDL-C, and HOMA-IR levels, indicating its superior efficacy in treating NAFLD. Notably, LGZG effectively regulates serum biochemical parameters critical for assessing NAFLD progression. Although GGT and LDL-C showed no significant differences between groups, this may stem from an insufficient number of cases, highlighting the need for further randomized controlled trials.

The observed efficacy in liver function markers (ALT, AST) and lipid reduction (TC, TG) may relate to the inhibition of TNF-α and Leptin expression, promoting hepatic metabolism and reducing inflammation [47]. LGZG may also mitigate hepatocyte damage by inhibiting hepatic Kupffer cells in the STING-TBK1-NF-kB pathway, decreasing inflammatory markers like TNF-α and IFN-β, thus alleviating liver inflammation and fat deposition [46].

## 5 LIMITATIONS AND SUGGESTIONS

### 5.1 Limitations

Despite promising results, this meta-analysis has limitations. While 12 studies reported random assignment, only 11 specified the randomization method. Most studies lacked detailed descriptions of blinding methods, with only one study providing specifics. This absence raises concerns about the potential exaggeration of efficacy due to less stringent methodological approaches. Additionally, publication bias may stem from including only studies conducted in China, and there was a lack of follow-up data and reports on adverse events, impacting the assessment of LGZG’s safety and efficacy.

### 5.2 Suggestions

To enhance the reliability of future studies, we recommend the following:

- **Prospective Registration:** Clinical trials should be registered prospectively, with well-designed protocols. Third-party supervision of allocation concealment and blinding is essential to ensure authenticity and improve literature quality.
- **Dialectic Treatment:** TCM treatments should be tailored based on specific patient needs guided by TCM principles to accurately assess efficacy.
- **Long-term Follow-up:** Extended follow-up is crucial for gathering patient data and evaluating long-term treatment effects, including documenting adverse events.

## CONCLUSION

Our findings suggests that LGZG effectively regulates lipids and protects liver function, making it a safe and effective alternative for NAFLD treatment. However, the effects on GGT and LDL-C remain uncertain, likely due to the limited number of cases. Given the methodological limitations of the retrieved studies, more rigorously designed, larger-scale, multicenter, double-blind, randomized controlled trials are necessary to validate the efficacy and safety of LGZG in treating NAFLD.

## Figures and Tables

**Table 1:** The composition of Ling Gui Zhu Gan Decoction and function of each herb.

Chinese Name	Pharmaceutical Name	Species	Family	Dosage	Function in Chinese Medicine
Fuling	Poria	*Poria cocos* (Schw.) Wolf	Polyporaceae	12 g	Promotes urination, leaches out dampness, fortifies the spleen and stomach, and calms the spirit
Guizhi	Cassiabarktree Twig	*Cinnamomum cassia Presl*	Lauraceae	9 g	Promotes sweating, resolves the flesh, warms and frees the channels and vessels, assists yang in transforming qi, downbeats qi
Baizhu	Atractylodis Macrocephalae Rhizoma	*Atractylodes macrocephala Koidz*	Compositae	6 g	Fortifies the spleen, boosts qi, dries dampness, promotes urination, stops sweating, calms the fetus
Gancao	Liquorice Root	*Glycyrrhiza uralensis Fisch*	Fabaceae	6 g	Supplements the spleen, boost qi, clears heat, resolves toxins, dispels phlegm, relieves cough, relaxes tension, relieves pain, harmonizes the nature of other medicinals

**Table 2:** Search strategy.

#1 Non-alcoholic fatty liver disease
#2 Non-alcoholic fatty liver
#3 Liver steatosis
#4 Non-alcoholic steatohepatitis
#5 #1 OR #2 OR #3 OR #4
#6 Lingguizhugan
#7 Lingguizhugan decoction
#8 Lingguizhugan formula
#9 #6 OR #7 OR #8
#10 Randomized controlled trial
#11 Controlled clinical trial
#12 Randomized
#13 Controlled
#14 Trial
#15 Random
#16 Placebo
#17 Groups
#18 Double-blind
#19 #10 OR #11 OR #12 OR #13 OR #14 OR #15 OR #16 OR #17 OR #18
#20 #5 AND #9 AND #19

**Table 3:** The characteristics of the included studies.

Author	Cases T/C	Age Range mean	Gender male/ female	The Course of Disease Range, mean	Intervention		Duration/ follow up	Adverse Events	Outcome Measures
					T	C			
Wen *et al.,* (2022) [30]	42/42	T 49.12, C 48.64	T 27/15, C 25/17	T 3.45y, C 3.39y	LGZG+Polyene phosphatidylcholine capsules	Polyene phosphatidylcholine capsules	3 months	NR	TER, TC, TG, LDL-C, HDL-C, ALT, AST, GGT
Luo *et al.,* (2022) [31]	50/50	T 32-64,43.8, C 30-62,43.5	T 31/19, C 30/20	T 4.72y, C 4.7y	LGZG+Polyene phosphatidylcholine capsules	Polyene phosphatidylcholine capsules	3 months	NR	TER, TC, TG, LDL-C, HDL-C, ALT, AST
Dong *et al.,* (2022) [32]	49/49	T 43.8, C 44.1	T 28/21, C 30/19	T 2.7y, C 2.5y	LGZG+Polyene phosphatidylcholine capsules	Polyene phosphatidylcholine capsules	8 weeks	NR	TER, TC, TG, ALT, AST
Chen *et al.,* (2021) [33]	40/40	T 45.9, C 46.3	T 22/18, C23/17	T 4.3y, C 3.8y	LGZG+Limit calories	Limit calories	4 weeks /3 months	NR	TER, TC, TG, ALT, AST, GGT
Shen *et al.,* (2020) [34]	30/30	T 25-63,43.1, C 24-65,42.86	T 16/14, C 15/15	T 3.14y, C 3.21y	LGZG+Silibinin Capsules	Silibinin Capsules	4 weeks	NR	TER, TG, ALT, AST
Xu *et al.,* (2020) [35]	81/81	T 57, C 56.67	T 34/47, C 34/47	T 5.18y, C 4.8y	LGZG	Placebo	12 weeks /4 weeks	NR	TC, TG, LDL-C, HDL-C, ALT, AST, GGT, HOMA-IR
Xue and Xie, (2020) [36]	51/49	T 22-66,45, C 21-65,43	T 28/23, C 26/23	T 1-3,2.9y, C 1-6,3.1y	LGZG+Polyene phosphatidylcholine capsules	Polyene phosphatidylcholine capsules	12 weeks	T 1 case with abdominal distension, 1 case with diarrhea, 2 cases with nausea, 1 case with vomiting, and 1 case with rash, C 2 cases with abdominal distension, 1 case with diarrhea, 2 cases with nausea, 1 case with vomiting, and 1 case with rash	TER, LDL-C, HDL-C, ALT, AST, GGT, HOMA-IR
Yu *et al.,* (2019) [37]	48/48	T 25-56,37.13, C 26-50,37.75	T 43/5, C 45/3	NR	LGZG+Bifid Triple Viable Capsules	Bifid Triple Viable Capsules	12 weeks /24 weeks	NR	TER, TC, TG, ALT
Sun and Wang, (2019) [38]	50/50	T 36-75,60.88, C 37-74,59.3	T 27/23. C 28/22	T 2-13,8.47y, C 2-12,7.47y	LGZG+Polyene phosphatidylcholine capsules	Polyene phosphatidylcholine capsules	20 days	NR	TER, TG, HOMA-IR
Song and Li, (2014) [39]	45/45	T 44.8, C 45.2	T 23/22, C 24/21	T 4.22y, C 4.2y	LGZG+Polyene phosphatidylcholine capsules	Polyene phosphatidylcholine capsules	3 months	NR	TER, ALT, AST
Song and Li, (2013) [40]	48/42	T 44.8, C 42.2	T 26/22, C 22/20	T 4.24y, C 4.2y	LGZG+Polyene phosphatidylcholine capsules	Polyene phosphatidylcholine capsules	3 months	NR	TER, TC, TG, ALT, AST
Li, (2011) [41]	36/36	T 42.21, C 40.27	T 22/14, C 20/16	T 3.01y, C 3.35y	LGZG	Hugan tablets+zhibituo tablets (Chinese patent medicine)	8 weeks	NR	TER, TC, TG, ALT, AST

## Data Availability

All the data and supportive information provided within the article.

## LIST OF ABBREVIATIONS

ALT: Alanine Aminotransferase
CBM: Chinese Biomedicine Database
CNKI: China National Knowledge Infrastructure
NAFLD: Non-Alcoholic Fatty Liver Disease
NASH: Non-Alcoholic Steatohepatitis
FDA: Food and Drug Administration
GGT: Glutamyl Transpeptidase
GLP-1RA: Glucagon-Like Peptide-1 Eeceptor Agonists
HOMA-IR: Homeostatic Model Assessment of Insulin Resistance
PRISMA: Preferred Reporting Items for Systematic Reviews and Meta-Analyses
SGLT2: Selective Sodium-Dependent Glucose Co-Transporter
TC: Total Cholesterol
TCM: Traditional Chinese Medicine
TER: Total Effective Rate
TG: Triglycerides

## STANDARDS OF REPORTING

PRISMA guidelines and methodology were followed.

## SUPPLEMENTARY MATERIAL

PRISMA checklist is available as supplementary material on the publisher’s website along with the published article.
