# Supplementary material for: Lingguizhugan Decoction in the Treatment of Non-Alcoholic Fatty Liver Disease: A Systematic Review and Meta-Analysis
Source: Endocr Metab Immune Disord Drug Targets. 2025 Jan 9;25(13):1060–72. doi: 10.2174/0118715303323071241022053842 (PMC12715390; doi:10.2174/0118715303323071241022053842)
Supplement: Supplementary file 1 — PRISMA checklist is available as supplementary material on the publisher’s website along with the published article. [file EMIDDT-25-13-1060_SD1.pdf]

# Supplementary Material

# Lingguizhugan Decoction in the Treatment of Non-Alcoholic Fatty Liver Disease: A Systematic Review and Meta-Analysis

Yifan Lu[1, 2, 3, #], Lijuan Nie[2, 3], Xinyi Yang[2, 3], Ziming Zhao[1], Yuxiao Wang[1], Qibiao Wu[1, *] and

Xiqiao Zhou[2, 3,]

*[1]State Key Laboratory of Quality Research in Chinese Medicines, Faculty of Chinese Medicine, Macau University of Science and Technology, Macau, P.R. China; [2]The First Clinical Medical College of Nanjing University of Chinese Medicine, Nanjing City, Jiangsu Province, 210000, China; [3]Department of Endocrinology, Jiangsu Province Hospital of Chinese Medicine, Affiliated Hospital of Nanjing University of Chinese Medicine, Nanjing City, Jiangsu Province, 210000, China*

## PRISMA 2020 Checklist

| Section and Topic | Item # | Checklist item | Location where item is reported |
|---|---|---|---|
| **TITLE** | | | |
| Title | 1 | The report is identified as a systematic review and meta-analysis. | Page.1 |
| **ABSTRACT** | | | |
| Abstract | 2 | The abstract includes Objective, Materials and Methods, Results, and Conclusion. | Page.1-2 |
| **INTRODUCTION** | | | |
| Rationale | 3 | Described in the Introduction. | Page.3-8 |
| Objectives | 4 | Started in the Introduction. | Page.3-8 |
| **METHODS** | | | |
| Eligibility criteria | 5 | Described in the Inclusion and Exclusion criteria. | Page.9 |
| Information sources | 6 | Described in the Search strategy. | Page.9 |
| Search strategy | 7 | Presented in the Search strategy. | Page.9 |
| Selection process | 8 | Described in the Flow diagram of literature screening. | Page.37 |
| Data collection process | 9 | Described in the Data extraction and risk of bias assessment. | Page.9-10 |
| Data items | 10a | Described in the Inclusion criteria. | Page.9 |
| | 10b | Described in the Inclusion criteria. | Page.9 |
| Study risk of bias assessment | 11 | Described in the Data extraction and risk of bias assessment. | Page.9-10 |
| Effect measures | 12 | Described in the Statistical analysis. | Page.10 |
| Synthesis methods | 13a | Described in the Table 3. | Page.30-36 |
| | 13b | Described in the Statistical analysis. | Page.10 |
| | 13c | Described in the Statistical analysis. | Page.10 |

| Section and Topic | Item # | Checklist item | Location where item is reported |
|---|---|---|---|
| | 13d | Described in the Statistical analysis. | Page.10 |
| | 13e | Described in the Statistical analysis. | Page.10 |
| | 13f | Described in the Statistical analysis. | Page.10 |
| Reporting bias assess-ment | 14 | Described in the Statistical analysis. | Page.10 |
| Certainty as-sessment | 15 | Described in the Statistical analysis. | Page.10 |
| **RESULTS** | | | |
| Study selec-tion | 16a | Described in the Characteristics of included trials and Flow diagram of literature screening. | Page.11, Page.37 |
| | 16b | Cited in the Characteristics of included trials and Flow diagram of literature screening. | Page.11, Page.37 |
| Study charac-teristics | 17 | Cited in the Characteristics of included trials. | Page.11 |
| Risk of bias in studies | 18 | Presented in the Outcome measures. | Page.12-14 |
| Results of individual studies | 19 | Presented in the Outcome measures and Fig. 4-9. | Page.12-14 |
| Results of syntheses | 20a | Presented in the Outcome measures and Fig. 4-9. | Page.12-14 |
| | 20b | Presented in the Outcome measures and Fig. 4-9. | Page.12-14 |
| | 20c | Presented in the Outcome measures and Fig. 4-9. | Page.12-14 |
| | 20d | Presented in the Outcome measures and Fig. 4-9. | Page.12-14 |
| Reporting biases | 21 | Presented in the Publication bias and Fig. 10. | Page.15 |
| Certainty of evidence | 22 | Presented in the Outcome measures and Fig. 4-9. | Page.12-14 |
| **DISCUSSION** | | | |
| Discussion | 23a | Described in the Discussion. | Page.15-17 |
| | 23b | Discussed in the Limitations. | Page.17-18 |
| | 23c | Discussed in the Limitations. | Page.17-18 |
| | 23d | Discussed in the Suggestions and Conclusions. | Page.18-19 |
| **OTHER INFORMATION** | | | |
| Registration and protocol | 24a | Provided in the Abstract. | Page.1-2 |
| | 24b | Described in the CRediT authorship contribution statement. | Page.19 |
| | 24c | Described in the Abstract. | Page.1-2 |
| Support | 25 | Described in the Funding. | Page.19 |
| Competing interests | 26 | Declared in the Conflicts of interest. | Page.19 |

| Section and Topic | Item # | Checklist item | Location where item is reported |
|---|---|---|---|
| Availability of data, code and other materials | 27 | Reported in the Annexed materials. | Page.27-43 |

*From:* Page MJ, McKenzie JE, Bossuyt PM, Boutron I, Hoffmann TC, Mulrow CD, et al. The PRISMA 2020 statement: an updated guideline for reporting systematic reviews. BMJ 2021;372:n71. doi: 10.1136/bmj.n71
For more information, visit: http://www.prisma-statement.org/
